# Effect of Antifibrotic MicroRNAs Crosstalk on the Action of N-acetyl-seryl-aspartyl-lysyl-proline in Diabetes-related Kidney Fibrosis

**DOI:** 10.1038/srep29884

**Published:** 2016-07-18

**Authors:** Swayam Prakash Srivastava, Sen Shi, Megumi Kanasaki, Takako Nagai, Munehiro Kitada, Jianhua He, Yuka Nakamura, Yasuhito Ishigaki, Keizo Kanasaki, Daisuke Koya

**Affiliations:** 1 Department of Diabetology & Endocrinology, Kanazawa Medical University, Uchinada, Ishikawa 920-0293, Japan; 2 Division of Anticipatory Molecular Food Science and Technology, Kanazawa Medical University, Uchinada, Ishikawa 920-0293, Japan; 3 Medical Research Institute, Kanazawa Medical University, Uchinada, Ishikawa 920-0293, Japan

## Abstract

N-acetyl-seryl-aspartyl-lysyl-proline (AcSDKP) is an endogenous antifibrotic peptide. We found that suppression of AcSDKP and induction of dipeptidyl peptidase-4 (DPP-4), which is associated with insufficient levels of antifibrotic microRNA (miR)s in kidneys, were imperative to understand the mechanisms of fibrosis in the diabetic kidneys. Analyzing streptozotocin (STZ)-induced diabetic mouse strains, diabetic CD-1 mice with fibrotic kidneys could be differentiated from less-fibrotic diabetic 129Sv mice by suppressing AcSDKP and antifibrotic miRs (miR-29s and miR-let-7s), as well as by the prominent induction of DPP-4 protein expression/activity and endothelial to mesenchymal transition. In diabetic CD-1 mice, these alterations were all reversed by AcSDKP treatment. Transfection studies in culture endothelial cells demonstrated crosstalk regulation of miR-29s and miR-let-7s against mesenchymal activation program; such bidirectional regulation could play an essential role in maintaining the antifibrotic program of AcSDKP. Finally, we observed that AcSDKP suppression in fibrotic mice was associated with induction of both interferon-γ and transforming growth factor-β signaling, crucial molecular pathways that disrupt antifibrotic miRs crosstalk. The present study provides insight into the physiologically relevant antifibrotic actions of AcSDKP via antifibrotic miRs; restoring such antifibrotic programs could demonstrate potential utility in combating kidney fibrosis in diabetes.

Diabetic nephropathy is a renal complication of diabetes and the leading cause of end-stage renal disease worldwide^1^. The final consequence of kidney diseases, including diabetic nephropathy, is kidney fibrosis^2^. The source and heterogeneity of kidney fibroblasts remain unclear^3^. N-acetyl-seryl-aspartyl-lysyl-proline (AcSDKP) is a tetrapeptide that is normally present in human plasma, and it is exclusively hydrolyzed by angiotensin-converting enzyme (ACE). ACE-I treatment increases the plasma level of AcSDKP fivefold^4^. Earlier studies supported the antifibrotic activity of AcSDKP (e.g., by inhibiting transforming growth factor (TGF)-β-induced expression of plasminogen activator inhibitor-1 and α2 collagen type I in human mesangial cells by inhibiting the smad 2/3 signaling pathway^5^. AcSDKP prevented mesangial matrix expansion in diabetic db/db mice^6^, and AcSDKP demonstrated protective effects on organs fibrosis in various experimental animal models with fibrosis^7^,^8^,^9^,^10^,^11^,^12^,^13^,^14^. Recently, we reported that AcSDKP prevents renal fibrosis by counteracting the endothelial mesenchymal transition (EndMT)^15^. Matrix-producing mesenchymal cells derived from EndMT contribute to the pathogenesis of kidney fibrosis^16^,^17^,^18^,^19^,^20^.

The enzyme activity of dipeptidyl peptidase-4 (DPP-4) is highest in the kidney per organ weight^21^. Such high level of DPP-4 in kidney could be involved in renal metabolism, physiology and disease development^21^,^22^,^23^. We have reported that DPP-4 could be a key molecule associated with kidney fibrosis, as well as with the inhibition of DPP-4 through linagliptin ameliorated kidney fibrosis associated with the inhibition of EndMT and TGF-β/smad signaling^18^.

The actions and synthesis of microRNA (miR)s are tightly regulated. Key antifibrotic miRs i.e. miR-let-7s and miR-29s suppression are important for understanding fibrotic mechanism in the diabetic kidney^15^,^18^,^24^,^25^. We have shown that AcSDKP inhibits kidney fibrosis by inducing miR-let-7s; DPP-4 inhibitor linagliptin ameliorates kidney functions by inducing miR-29s in the same model^15^,^18^. These molecules exhibited similar antifibrotic mechanisms, such as anti-mesenchymal activity and anti-TGF-β/smad signaling pathways. Therefore, we hypothesized that there was potential crosstalk between AcSDKP and DPP-4 inhibition through antifibrotic miRs crosstalk.

## Results

### Fibrotic kidney mouse strain exhibited the induction of DPP-4, the suppression of AcSDKP and low antifibrotic miRs levels in diabetes

**Figure 1:**
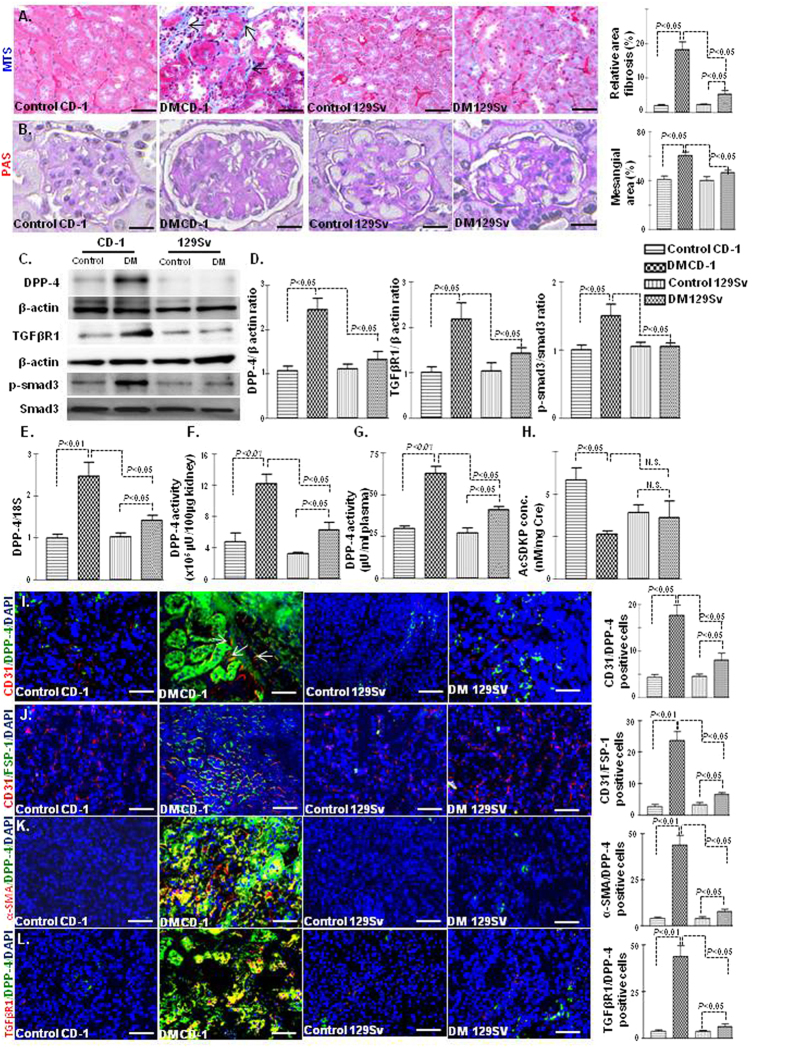
Fibrotic kidneys exhibited the induction of DPP-4 and the suppression of AcSDKP in diabetes. (**A**) Masson’s trichrome staining (MTS) in the non-diabetic and diabetic kidneys of CD-1 and 129Sv mice strains and the quantification of the relative area fibrogenesis (RAF). Scale bar: 50 μM. (**B**) Periodic acid–Schiff (PAS) staining and the quantification of relative mesangial area in glomerular tufts (GSA). Scale bar: 50 μM. (**C**) Western blot analysis of DPP-4, TGFβR1 and smad3 phosphorylation in the non-diabetic and diabetic kidneys of the CD-1 and 129Sv mice strains. Representatives from 5 blots are shown here. (**D**) Densitometry analysis of DPP-4, TGFβR1, and smad3 phosphorylation. DPP-4 and TGFβR1 were normalized by β-actin, smad3 phosphorylation was normalized by total-smad3. (**E**) Gene expression analysis of DPP-4 by qPCR in the non-diabetic and diabetic kidney. N = 4 were analyzed in control group while N = 6 were analyzed in diabetic group in each strain (**F**) DPP-4 activity analysis in kidney. (**G**) DPP-4 activity analysis in the plasma. N = 5 were analyzed in each group. (**H**) AcSDKP concentration in the urine; urine AcSDKP levels were normalized by the urine creatinine level. N = 5 were analyzed in control while N = 6 were analyzed in diabetic group. (**I**) Immunofluorescence analysis of CD31/DPP-4 in the kidneys of the non-diabetic and diabetic mice of CD-1 and 129Sv strains. Scale bar: 50 μM. (**J**) Immunofluorescence analysis of CD31/FSP-1 in the kidneys of the non-diabetic and diabetic 129Sv mice. CD31 red-rhodamin, FSP-1 green-FITC. Scale bar: 50 μM in. (**K**) Immunofluorescence analysis of α-SMA/DPP-4 in the kidneys of the non-diabetic and diabetic mice of CD-1 and 129Sv mice strains. α-SMA red-rhodamin, DPP-4 green-FITC. Scale bar: 50 μM. (**L**) Immunofluorescence analysis of TGFβR1/DPP-4 in the kidneys of the non-diabetic and diabetic mice of CD-1 and 129Sv mice strains. TGFβR1 red-rhodamin, DPP-4 green-FITC. Scale bar: 50 μM. Merged pictures are shown. N = 5 were analyzed in each data set. The data are expressed as the means ± s.e.m and are included in the graph. The diabetic mice are designated as DM in the figure.

In mice, the kidney fibrosis phenotype is largely dependent upon the strain specificity^26^. We analyzed comparative renal histopathology in non-diabetic and streptozotocin (STZ)-induced diabetic CD-1 and 129Sv mice. At the time of sacrifice, the diabetic CD-1 and diabetic 129Sv mice had similar blood glucose levels (Fig. S1A), reduced body weight (Fig. S1B), and higher kidney weight per body weight (Fig. S1C) compared with those of the non-diabetic animals. The kidneys of the CD-1 mice showed fibrosis, with excessive deposition of extracellular matrix while the 129Sv strain displayed minor fibrotic alterations (Fig. 1A). PAS staining revealed increased mesangial areas (Fig. 1B) in the kidneys of diabetic CD-1 mice compared with those of the diabetic 129Sv mice. The plasma of diabetic CD-1 mice exhibited elevated level of cystatin C whereas such alteration in cystatic C level was not found in diabetic 129Sv when compared to control (Fig. S1D). Western blot analysis revealed that the protein expression of DPP-4 and TGFβR1 and smad3 phosphorylation were elevated in the kidneys of the diabetic CD-1 mice while such kind of alteration was not found in the kidneys of diabetic 129Sv mice when compared with those of the non-diabetic mice (Fig. 1C,D). In addition, the diabetic CD-1 mice displayed higher DPP-4 gene expression levels (Fig. 1E). DPP-4 enzymatic activity in either the kidney or plasma was significantly higher in the diabetic CD-1 mice than in the control mice; minor induction of DPP-4 activity in diabetes was also observed in the 129Sv strain, although it was significantly lower than in the CD-1 mice (Fig. 1F,G). When analyzing the antifibrotic peptide AcSDKP levels in the urine, diabetic CD-1 mice displayed significantly lower AcSDKP levels in the urine than the controls, whereas such diabetes-induced alterations in the AcSDKP levels were not found in the 129Sv mice (Fig. 1H). Such difference in AcSDKP level in strains was not explained by known mechanisms (Fig. S1E–I).

We further analyzed the implications of inducing the expression of DPP-4 and its association with EndMT in the diabetic kidney. Remarkably, higher CD31/DPP-4 immunolabeling was observed in the kidneys of the diabetic CD-1 mice than in those of the diabetic 129Sv mice (Fig. 1I). Increased co-immunolabeling of CD31/FSP-1 (Fig. 1J), CD31/α-SMA (Fig. S1J), CD31/TGFβR1 (Fig. S1K), α-SMA/DPP-4 (Fig. 1K),TGFβR1/DPP-4 (Fig. 1L) and FSP-1/DPP-4 (Fig. S1L) in diabetic CD-1 mice suggested that fibrotic changes in the CD-1 mice were associated with the induction of DPP-4, EndMT and activation of TGF-β/smad signaling on endothelial cells.

**Figure 2:**
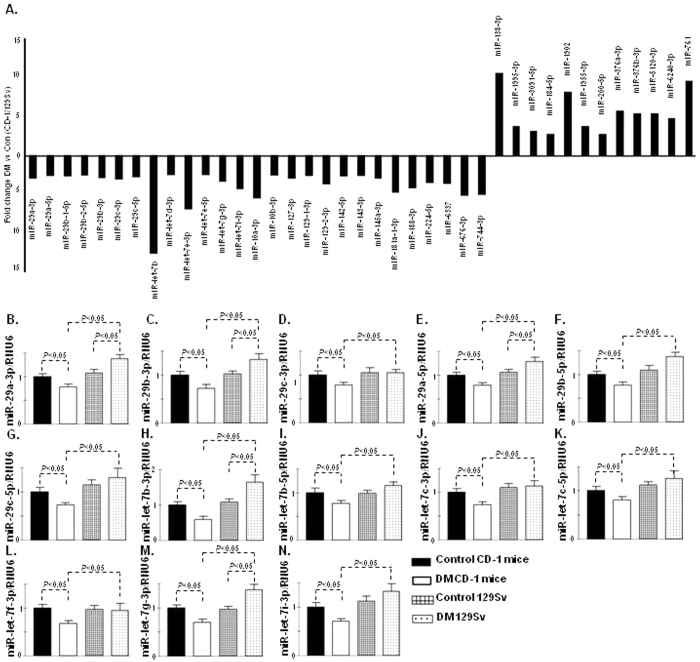
MiR-29s and miR-let-7s reveal similar trends of suppression in the kidneys of the diabetic CD-1 mice but not in the 129Sv mice. (**A**) miRs alteration in the kidneys of the diabetic and non-diabetic CD-1 and 129Sv mice, as analyzed by microRNA array analysis. N = 3 in case of control kidneys while N = 3 were analyzed in diabetic kidneys of each mouse strain. (**B**) qPCR analysis of miR-29a-3p. (**C**) miR-29b-3p. (**D**) miR-29c-3p. (**E**) miR-29a-5p. (**F**) miR-29b-5p. (**G**) miR-29c-5p. (**H**) miR-let-7b-3p. (**I**) miR-let-7b-5p. (**J**) miR-let-7c-3p. (**K**) miR-let-7c-5p. (**L**) miR-let-7f-3p. (**M**) miR-let-7g-3p. (**N**) miR-let-7i-3p. N = 5 were analyzed in each data set. The data are expressed as the means ± s.e.m and are included in the graph. Hs_RNU6 was used as internal control. Diabetic mice are designated as DM in the figure.

To evaluate the miR expression signature in the fibrotic kidney of STZ-induced diabetic mice, we performed miRNA microarray analysis in the kidneys of CD-1 and 129Sv in both non-diabetic and diabetic mice. Sixty-two miRs were downregulated, and 30 were upregulated in diabetic CD-1 mice, whereas 26 miRs were downregulated, and 61 miRs were upregulated in the diabetic 129Sv mice strains compared with the non-diabetic control mice (data shown in excel file). Among these miRs, we found that 27 miRs were downregulated, and 12 were upregulated in the diabetic CD-1 mice compared with the diabetic 129Sv mice (Fig. 2A). MiR-29s and let-7s emerged as major altered miRs and showed a remarkable trend of suppression in the diabetic CD-1 mice; such alteration was not found in the case of the 129Sv diabetic mice (Fig. 2A). Validation of miR-29s and miR-let-7s by qPCR revealed similar trends of suppression in the diabetic CD-1 mice, and there were no such alterations in the diabetic 129Sv mice compared with the corresponding non-diabetic mice (Fig. 2B–N).

### AcSDKP inhibited TGF-β-stimulated DPP-4 and induced anti-fibrotic miRome

**Figure 3:**
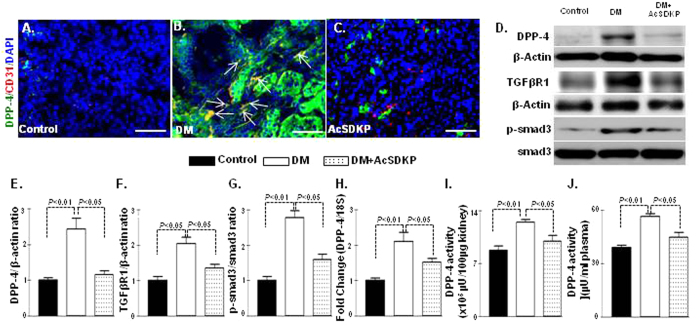
AcSDKP inhibits DPP-4-associated TGF-β signaling in the kidneys of the diabetic mice. (**A–C**) Immunofluorescence analysis in the kidneys of control mice, DM mice and DM mice treated with AcSDKP. Representative pictures are shown. Scale bar: 50 μM in each panel. Arrows indicate co-immunolabelling. Merged pictures are shown. N = 4 were analyzed. (**D**) Western blot analysis of DPP-4, TGFβR1 and smad3 phosphorylation in the kidneys of the control, diabetic and AcSDKP-treated mice. Representatives from 5 blots are shown here. (**E–G**) Densitometry quantification of DPP-4, TGFβR1 and smad3 phosphorylation. The data were normalized by β-actin for DPP-4 and TGFβR1. Phospho smad3 was normalized by total smad3. N = 5 were analyzed. (**H**) qPCR analysis of DPP-4 mRNA expression. N = 5 in control while N = 6 in case of diabetic and AcSDKP treated diabetic kidneys were analyzed (**I**) DPP-4 activity analysis in kidney homogenate. N = 5 were analyzed in each group. (**J**) DPP-4 activity analysis in the plasma. N = 5 were analyzed in each data set. The data are expressed as the means ± s.e.m and are included in the graph. Diabetic mice are designated as DM in the figure.

**Figure 4:**
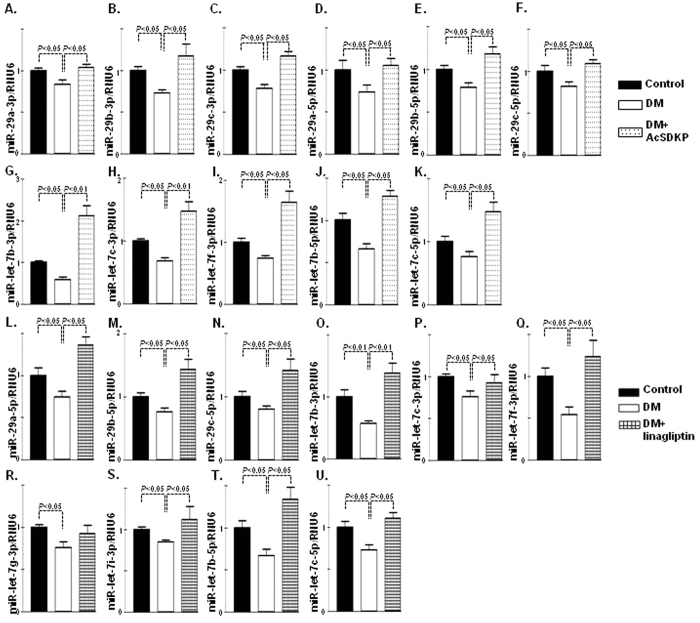
Upregulation of antifibrotic miR-29s and miR-let-7s in AcSDKP or DPP-4 inhibitor-treated diabetic kidney. (**A–K**) miR-29s and miR-let-7s emerged as important regulatory antifibrotic molecules, as validated by real-time PCR using specific primers in the kidneys of the control, diabetic and AcSDKP treatment. (**A**) qPCR analysis of miR-29a-3p. (**B**) miR-29b-3p. (**C**) miR-29c-3p. (**D**) miR-29a-5p. (**E**) miR-29b-5p. (**F**) miR-29c-5p. (**G**) miR-let-7b-3p. (**H**) miR-let-7c-3p. (**I**) miR-let-7f-3p. (**J**) miR-let-7b-5p. (**K**) miR-let-7c-5p. N = 5 were analyzed. (**L–U**) qPCR analysis in the kidneys of control, diabetic and linagliptin-treated mice (**L**) miR-29a-5p. (**M**) miR-29b-5p. (**N**) miR-29c-5p. (**O**) miR-let-7b-3p. (**P**) miR-let-7c-3p. (**Q**) miR-let-7f-3p. (**R**) miR-let-7g-3p. (**S**) miR-let-7i-3p. (**T**) miR-let-7b-5p. (**U**) miR-let-7c-5p. N = 5 in case of control whereas N = 6 in case of diabetic and linagliptin treated diabetic kidneys were analyzed. Hs_RNU6 was used as internal control to normalize the data. The data are expressed as the means ± s.e.m and are included in the graph. Diabetic mice are designated as DM in the figure.

High DPP-4 levels have shown to be associated with kidney fibrosis in diabetic CD-1 mice and also with the induction of mesenchymal activation program^18^,^27^. AcSDKP administration significantly suppressed CD31/DPP-4 (Fig. 3A–C), α-SMA/DPP (Fig. S2A), FSP-1/DPP-4 (Fig. S2B), TGFβR1/DPP-4 (Fig. S2C) and protein/mRNA levels of DPP-4 in the diabetic kidneys of CD-1 mice (Fig. 3D–H). AcSDKP treatment in the diabetic mice also showed reduced protein levels of TGFβR1 and smad3 phosphorylation compared with the kidneys of the diabetic mice (Fig. 3D,F,G). DPP-4 activities in both plasma and kidneys exhibited higher levels in diabetic mice; AcSDKP treatment significantly reduced the DPP-4 activity (Fig. 3I–J). AcSDKP administration also suppressed the plasma cystatin C levels in diabetic CD-1 mice (Fig. S2D). In the Human Dermal Microvascular Endothelial Cells (HMVEC), the TGF-β2 treatment increased the DPP-4, TGFβR1 protein levels and the smad3 phosphorylation; AcSDKP treatment decreased these levels (Fig. S3A–D). The AcSDKP treatment also reduced the DPP-4 mRNA levels in the TGF-β2-stimulated HMVECs (Fig. S3E). In our previous studies we have shown that miR-29s target DPP-4 by binding on the 3′UTR of DPP-4 mRNA^18^. In this study we analyzed the luciferase activity in transfected HMVECs carrying the construct containing the 3′UTR of the DPP-4 mRNA^18^; we observed that the TGF-β2 treatment increased the luciferase activity, whereas AcSDKP treatment significantly reduced the TGF-β2-induced luciferase activity in the HMVECs (Fig. S3F), suggesting that AcSDKP inhibited DPP-4 transcription in a 3′-UTR dependent manner. Therefore, we analyzed the miR-expression profile of the kidneys of the AcSDKP-treated diabetic mice and found that the AcSDKP-treated kidneys of the diabetic mice exhibited significant restoration of miR-29s and miR-let-7s expression levels compared with the diabetic mice (Fig. 4A–K). *In vitro*, the stimulation by TGF-β2 on the HMVECs decreased the miR-29s expression level, and the AcSDKP treatment markedly increased the miR-29s expression levels (Fig. S4A–C).

### DPP-4 inhibition also induced anti-fibrotic miRome

In the series of attempts studying the effect of DPP-4 inhibition on anti-fibrotic miRs, we also analyzed the expression levels of miR-29s (miR-29a, miR-29b and miR-29c) and miR-let-7s (miR-let-7b, miR-let-7c, miR-let-7f, miR-let-7g and miR-let-7i) in the kidneys of diabetic mice and linagliptin-treated diabetic mice. We found that miR-29s and miR-let-7s were significantly reduced in the diabetic group compared with those in the control group and that linagliptin treatment caused upregulation in miR-29s and miR-let-7s (miR-let-7b, miR-let-7c, miR-let-7f, miR-let-7g and miR-let-7i) compared with the kidneys of diabetic mice (Fig. 4L–U). Linagliptin treatment in the TGF-β2-induced HMVECs also showed upregulation in the miR-let-7s (Fig. S4D–I). We have recently shown that miR-29s negatively regulate DPP-4 and that inhibition of DPP-4 with linagliptin triggered upregulated miR-29s gene expression, which was associated with the inhibition of mesenchymal activation program and renal fibrosis^18^.

### Bidirectional regulation of miR-29s and miR-let-7s in HMVECs

**Figure 5:**
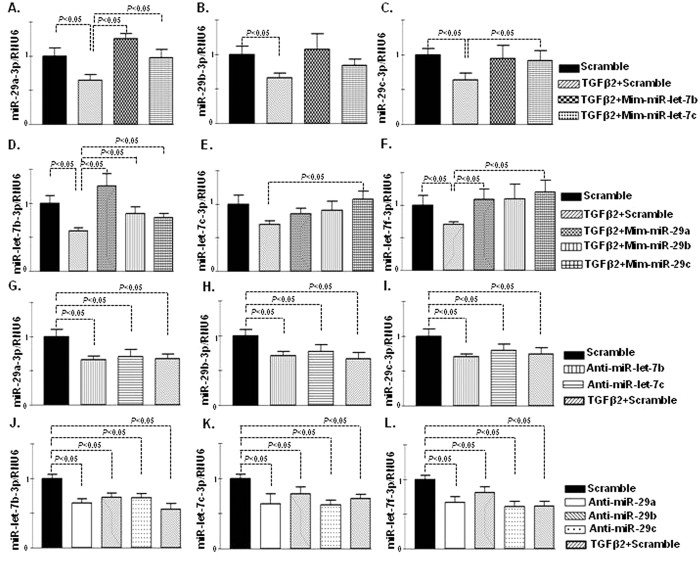
Bidirectional regulation of miR-let-7s (b and c) and miR-29s (a, b and c) in HMVECs. (**A–C**) Gene expression studies by qPCR analysis of miR-29s after transfection of scramble control and mimetic constructs (mim-miR-let-7b and mim-miR-let-7c) in the presence of TGFβ2 in the HMVECs. (**A**) miR-29a-3p. (**B**) miR-29b-3p. (**C**) miR-29c-3p. N = 5 were analyzed in data set. (**D–F**) Gene expression studies by qPCR analysis of miR-let-7s after transfection of scramble control and mimetic constructs (mim-miR-29a, mim-miR-29b and mim-miR-29c) in the presence of TGFβ2 in the HMVECs. (**D**) miR-let-7b-3p (**E**) miR-let-7c-3p. (**F**) miR-let-7f-3p. N = 6 were analyzed in data set. (**G–I**) Gene expression analysis of miR-29s after the transfection of scramble control, antagomirs (anti-miR-let-7b and anti-miR-let-7c) and TGFβ2 treated scramble control in the HMVECs. (**G**) miR-29a-3p (**H**) miR-29b-3p. (**I**) miR-29c-3p. N = 5 were analyzed in each data set. (**J-L**) Gene expression analysis of miR-let-7s after the transfection of scramble control, antagomirs (anti-miR-29a, anti-miR-29b and anti-miR-29c) and TGFβ2 treated scramble control in the HMVECs. (**J**) miR-let-7b-3p. (**K**) miR-let-7c-3p. (**L**) miR-let-7f-3p. N = 6 were analyzed in each data set. Hs_RNU6 was used as internal control. The data are expressed as the means ± s.e.m and are included in the graph.

Antifibrotic interventions, such as AcSDKP and DPP-4 inhibitor linagliptin, induced similar anti-fibrotic miRome *in vivo* and *in vitro*. These data led us to confirm the crosstalk of anti-fibrotic miRs. MiR-let-7b and miR–let-7c were predominant miR displaying anti-EndMT^28^. Overexpression of miR-let-7b and miR-let-7c by mimic treatment (mim-miR-let-7b and mim-miR-let-7c) in HMVECs in the presence of TGF-β2 induced the expression of miR-29s (Fig. 5A–C). The miR-29a-3p was remarkably higher in the mim-miR-let-7b- and mim-miR-let-7c-transfected cells (Fig. 5A), whereas miR-29b-3p demonstrated a higher trend in the cells transfected with mim-miR-let-7s (Fig. 5B). MiR-29c-3p exhibited an increased trend in the mim-miR-let-7c transfected cells (Fig. 5C). Similarly, overexpression of miR-29s by mimic treatment (mim-miR-29a, mim-miR-29b and mim-miR-29c) in the presence of TGF-β2 increased the expression of miR-let-7s (Fig. 5D–F). On the contrary, the inhibition of miR-let-7s by antagomir (anti-miR-let-7b and anti-miR-let-7c) treatment reduced the levels of miR-29s (Fig. 5G–I), and the inhibition of miR-29s by antagomir treatment (i.e., anti-miR-29a, anti-miR-29b and anti-miR-29c) again reduced the level of miR-let-7s (Fig. 5J–L).

### Interferon-γ disrupts anti-fibrotic miRs crosstalk

**Figure 6:**
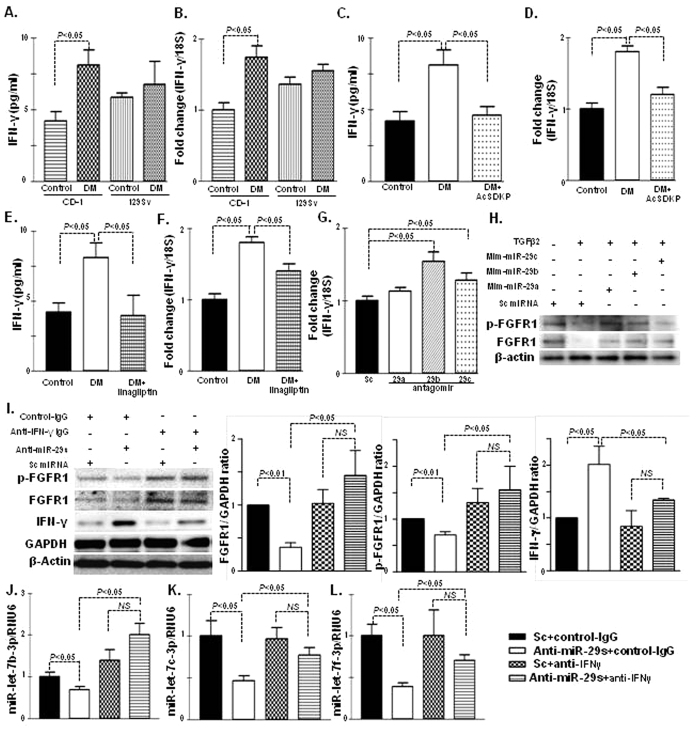
Suppression of IFN-γ and upregulated level of FGF1-phosphorylation by AcSDKP mediates crosstalk bidirectional regulation of miR-29s and miR-let-7s. (**A**) IFN-γ level determination in the control and DM of CD-1 and 129Sv mice strains, n = 5 were analyzed in each data set. (**B**) Gene expression of IFN-γ mRNA expression level in kidney of the control and DM of the CD-1 and 129Sv strains, n = 4 were analyzed in each data set. (**C**) IFN-γ level in the plasma of control, DM and AcSDKP-treated diabetic mice, n = 5 were analyzed in each data set. (**D**) qPCR analysis of IFN-γ mRNA gene expression in the kidneys of the control, DM and AcSDKP-treated diabetic kidney, n = 5 were analyzed in each data set. (**E**) IFN-γ level in the plasma of control, DM and linagliptin treated diabetic mice, n = 5 were analyzed in each data set. (**F**) qPCR analysis of IFN-γ mRNA gene expression in the kidneys of the control, DM and linagliptin-treated diabetic kidneys, n = 6 were analyzed in each data set. (**G**) qPCR gene expression analysis of IFN-γ mRNA level in the HMVECs transfected with antagomirs of miR-29s, n = 6 were analyzed in each data set. (**H**) Western blot analysis of the mimic treatment of miR-29s in the HMVECs. Representatives from 5 blots are shown here. (**I**) Western blot analysis of the antagomir treatment of miR-29s in the HMVECs, with or without neutralizing antibody for IFN-γ antibody and quantification of densitometric analysis. Representative from 5 blots are shown here. (**J–L**) qPCR analysis of miR-let-7s after transfection of the antagomir treatment of miR-29s in the HMVECs, with or without neutralizing IFN-γ antibody, n = 6 were analyzed in each data set. Hs_RNU6 was used as internal control. The data are expressed as the means ± s.e.m and are included in the graph.

Inflammation is the key factor during the fibroblast activation process^29^,^30^,^31^,^32^. The plasma of diabetic CD-1 mice exhibited elevated levels of interferon (IFN)-γ, the key inflammatory cytokine in fibrotic pathogenesis^32^, compared with the plasma of non-diabetic CD-1 mice; such an aberration was not found in the diabetic 129Sv strains (Fig. 6A). The IFN-γ gene expression was also induced in the kidneys of the diabetic CD-1 mice compared with those of the non-diabetic controls; such an aberration was not found in the diabetic kidneys of the 129Sv mice (Fig. 6B). AcSDKP treatment in the diabetic CD-1 mice significantly reduced the plasma IFN-γ (Fig. 6C) and mRNA levels in the kidneys (Fig. 6D). A similar suppression of the IFN-γ levels was observed in the kidneys of the diabetic CD-1 mice treated with DPP-4 inhibitor linagliptin (Fig. 6E,F).

MiR-29s target 3′UTR of IFN-γ mRNA and suppress IFN-γ levels^33^. Interestingly, IFN-γ has been shown to suppress both fibroblast growth factor receptor (FGFR1) and FGFR1-dependent miR-let-7s production^28^. Such suppression in miR-let-7s resulted in the induction of TGFβR1 and the promotion of EndMT^28^. Therefore, we analyzed whether FGFR1 expression levels affected the control of miR-29s. First, we reconfirmed the inhibitory role of miR-29s on IFN-γ expression in HMVECs (Fig. 6G). TGF-β2-suppressed both FGFR1 protein levels and phosphorylation of FGFR1; mim-miR-29s inhibited such TGF-β2-mediated alterations on FGFR1 (Fig. 6H). Finally, we analyzed the FGFR1 protein levels, phosphorylation of FGFR1, and miR-let-7s gene expression levels in the antagomirs of the miR-29s (combination of a, b, c)-treated HMVEC, with or without IFN-γ neutralizing antibody incubation. Interestingly, we observed that the suppression of FGFR1 protein levels and phosphorylation of FGFR1 induced by the antagomirs of miR-29s were restored by the IFN-γ neutralizing antibody incubation (Fig. 6I). In addition, the IFN-γ neutralizing antibody restored the levels of miR-29s antagomir-suppressed miR-let-7s levels in HMVEC (Fig. 6J–L). These data demonstrated that IFN-γ is a key profibrotic cytokine through the disruption of anti-fibrotic miRs crosstalk between miR-29s and miR-let-7s.

## Discussion

The physiological importance of AcSDKP has been reported elsewhere^15^,^34^,^35^,^36^. Suppression of AcSDKP in the fibrotic strain is associated with the induction of DPP-4, activation of TGF-β/smad signaling and down regulation of antifibrotic miRs (miR-29s and miR-let-7s), whereas such alterations were not prominent in the less-fibrotic diabetic 129Sv mice or in the diabetic CD-1 mice treated with an intervention of AcSDKP, thereby demonstrating the physiological significance of AcSDKP in fibrogenic programs in diabetic kidneys.

AcSDKP is degraded by the N-terminal catalytic domain of ACE. N-terminal truncation mutations of ACE in mice demonstrate protection from bleomycin-induced lung fibrosis and such anti-fibrotic effects phenotype in N-terminal truncation mutant mice was abolished when treated with S-17092, a prolyl-oligopeptidase inhibitor that inhibits the formation of AcSDKP^37^. Macconi *et al*.^36^, reported that high levels of miR-324-3p and the associated suppression of POP expression led to the suppression of low AcSDKP levels in urine and progressive renal damage in Munich Wistar Frömter (MWF) rats. Thymosin β4, the precursor peptide of AcSDKP, also exhibited anti-fibrotic and tissue repair effects^35^. POP inhibitor treatment combined with thymosin β4 administration abolished such beneficial effects of thymosin β4 and even displayed a pro-fibrotic phenotype^35^, demonstrating that in the metabolism of AcSDKP, POP is the critical enzyme for production and that ACE is the enzyme responsible for the degradation of AcSDKP. In our analysis, however, the suppression of AcSDKP in fibrotic diabetic CD-1 mice cannot be explained by either pathway because we found that POP activity was significantly higher and ACE activity/protein levels were reduced in fibrotic diabetic CD-1 mice. The levels of thymosin β4 were robust in both strains; therefore, there must be altered regulatory mechanisms other than either POP or ACE. However, currently there is no explanation available in the literature or from our experimental data set. Further studies would be required to investigate the detailed molecular regulations of AcSDKP.

Importantly, suppression of AcSDKP in diabetic CD-1 mice was associated with the induction of DPP-4 protein and enzyme activity. A profibrotic role of DPP-4 has been described by many researchers^18^,^21^,^22^,^23^. Elevated levels of DPP-4, TGF-β/smad signaling and downregulation of anti-fibrotic miRs in the fibrotic strain have been associated with increased EndMT in diabetes. After the restoration of AcSDKP levels in diabetic CD-1 mice, the mice displayed suppressed AcSDKP levels, inhibited renal fibrosis in fibrotic kidney involved concomitant reduction in the DPP-4-associated increase in the mesenchymal activation program, suggesting that the suppression of the endogenous antifibrotic peptide AcSDKP is a therapeutic target for combating kidney fibrosis.

**Figure 7:**
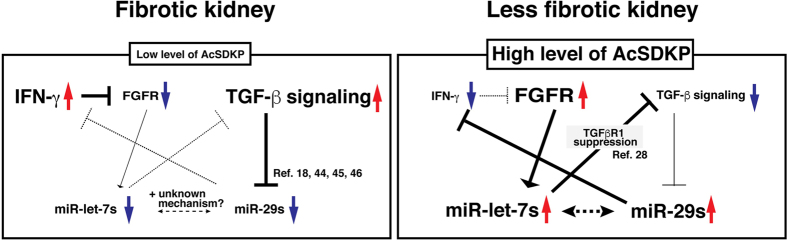
The working hypothesis of AcSDKP-mediated anti-fibrotic program through anti-fibrotic miRs crosstalk. AcSDKP could induce both miR-29s and miR-let-7s. Elevated level of miR-let-7s suppressed TGF-β associated signaling, which concomitantly induced the miR-29s gene expression. Elevated levels of miR-29s inhibit of IFN-γ, which in turn activates FGFR1 phosphorylation, which was essential for miR-let-7 induction. AcSDKP could regulate such anti-fibrotic miRs crosstalk.

Several reports have described the regulatory role of miRs in kidney disease and progression^19^,^24^,^38^,^39^,^40^,^41^,^42^. Recently TGF-β1 regulated crosstalk of microRNAs were found deregulated in type 1 diabetes patients who were at the risk of rapid progression to ESRD^43^. Interestingly, the miR-29s and miR-let-7s displayed crosstalk regulation, and AcSDKP induced such crosstalk of miR-29s and miR-let-7s in HMVECs. MiR-let-7s has been shown to inhibit TGFβR1^28^, and TGF-β/smad signaling has been well described as an inhibitory pathway of miR-29s^18^,^44^,^45^,^46^; therefore, it was not surprising to find that ‘miR-let-7s induced miR-29s’. However, a contrary pathway, molecular mechanisms of ‘miR-29s induced miR-let-7s expression’, could not be explained by a known direct molecular target of miR-29s. Thus, we focused on the involvement of the inflammatory cytokine IFN-γ. IFN-γ mRNA is the known target for miR-29s^33^, and we re-confirmed that observation. In addition, IFN-γ has been shown to inhibit FGFR1, a molecule that exhibits essential roles in the induction of miR-let-7s^28^. The suppressed miR-29s in the fibrotic kidneys resulted in the elevation of IFN-γ; subsequently, synthesized IFN-γ inhibited both FGFR1- and FGFR1-dependent miR-let-7s expression levels in diabetes. This suppression of miR-let-7 could induce TGFβR1 expression; again, activated TGF-β/smad signaling inhibited miR-29s^18^. These series of events could limit the crosstalk regulation between miR-29s and miR-let-7s during the fibrotic process in diabetic kidneys. Figure 7 depicts the involvement of AcSDKP in the homeostasis of anti-fibrotic milieu in the diabetic kidney. In short, elevated levels of miR-let-7s suppressed TGF-β-associated signaling, which concomitantly induced the miR-29s gene expression. Elevated levels of miR-29s inhibit IFN-γ, which in turn activates FGFR1 phosphorylation, finally suppressed TGF-β and associated signaling. These series of events finally leads to upregulation of miR-let-7s (Fig. 7). Therefore, miR-29s and miR-let-7s showed bidirectional crosstalk regulation. These data support the idea that AcSDKP is the key endogenous anti-fibrotic peptide responsible for the homeostasis of anti-fibrotic miRs in the kidneys.

In conclusion, AcSDKP-mediated antifibrotic program were contributing to the maintenance of the kidney homeostasis at least in part. The importance of anti-fibrotic peptide AcSDKP has been underestimated even when considering the biological significance of ACE inhibition on organ fibrosis. This study, therefore, provides novel insights into the understanding of miR-mediated alterations, which might be critical in the regulation of the anti-fibrotic program of AcSDKP.

## Materials and Methods

### Reagents and antibodies

AcSDKP was a gift from Dr. Omata from Asabio Bio Technology (Osaka, Japan). Linagliptin was provided by Boehringer Ingelheim (Ingelheim, Germany), with an MTA. The rat polyclonal anti-mouse CD31 antibody was purchased from EMFRET Analytics GmbH & Co. KG (Eibelstadt, Germany). The mouse monoclonal anti-β-actin, rabbit polyclonal anti-TGFβ-receptor I and goat polyclonal DPP-4 antibodies were obtained from Sigma (St. Louis, MO, USA). A rabbit polyclonal anti-phospho smad3 (s423 and s425) antibody was purchased from Rockland immunochemicals (Gilbertsville, PA). Mouse monoclonal anti-human CD31 and a goat polyclonal anti-mouse DPP-4 (for tissue labeling) were purchased from R&D System (Minneapolis, MN). A rabbit polyclonal anti-αSMA antibody was purchased from Gene Tex (Irvine, CA). A rabbit monoclonal anti-interferon gamma antibody and anti-TGFβ-receptor-II antibody were purchased from Abcam (Cambridge, UK). Anti-human interferon gamma antibody (neutralizing) was purchased from Affymetrix (Santa Clara, CA, US). Fluorescein-, rhodamine-, and Alexa 647-conjugated secondary antibodies were obtained from Jackson ImmunoResearch (West Grove, PA, USA). Anti-fibroblast specific proteins (FSP1, sometimes displayed as S100A4) and the HRP-conjugated secondary antibodies for western blot detection were purchased from Cell Signaling Technology (Danvers, MA, USA). Recombinant human TGF-β2 were purchased from PeproTech (Rocky Hill, NJ, USA).

### Animal experiments

The experiments in the methods sections are carried out in accordance with Kanazawa Medical University animal protocols (protocol number 2014-89; 2013-114 and 2014-101), approved by institutional animal care and use committee (IACUC). Authors confirm that all the experiments are performed in accordance to Japanese guidelines and regulations for scientific and ethical experimentation. The induction of diabetes in the CD-1 and 129Sv mice was performed according to the previously established experimental protocol^26^. In brief, 8-week-old CD-1 mice were induced diabetes with the single intraperitoneal injection of STZ at 200 mg/kg in 10 mmol/l citrate buffer (pH 4.5)^26^. For 129Sv mice, we injected 50 mg/kg of STZ via intraperitoneally for 5 consecutive days^26^. As the control set, citrate buffer without STZ was injected. Cystatin C levels in plasma were analyzed using the Mouse/Rat cystatin C kit (R&D System).

For the interventional study, we utilized a fibrotic diabetic kidney disease model (STZ-treated CD-1 mice). Sixteen weeks after the induction of diabetes, the diabetic mice were divided into the following two groups: a control group and an AcSDKP treatment group (500 μg/kg BW/day using an osmotic mini-pump) for 8 weeks^15^. In the second set of experiments, we utilized two groups of diabetic animals: a diabetic control (DM), and a linagliptin treatment group, which was treated with linagliptin (5 mg/kg BW/day) for 4 weeks as previously reported^18^.

### *In vitro* experiment

Human dermal microvascular endothelial cells (HMVECs, Lonza, Basel, Switzerland) cultured in EGM medium were used in this experiment. When the HMVECs on the adhesion reagent (Kurabo medical, Osaka, Japan) reached 70% confluence, 5 ng/ml recombinant human TGF-β2 for 48 h was placed in the experimental medium (HuMedia-MVG in serum-free RPMI at a 1:3 ratio), with or without AcSDKP (100 nM) preincubation for 2 h.

### Western blot analysis

Protein lysates were denatured in a SDS sample buffer at 100 °C for 5 min and were separated on SDS-polyacrylamide gels and blotted onto PVDF membranes (Pall Corporation, Pensacola, FL, USA) using the semidry method. The immunoreactive bands were developed using an enhanced chemiluminescence (ECL) detection system (Pierce Biotechnology, Rockford, IL, USA) and detected using an ImageQuant LAS 400 digital biomolecular imaging system (GE Healthcare Life Sciences, Uppsala, Sweden).

### DPP-4 activity detection

A DPP-4 activity Fluorometric assay kit was used for DPP-4 activity detection (Biovision, Milpitas, CA, USA). Activity was determined as per the manufacturer’s instructions. Enzyme activity was expressed in pmol/min/ml (μU/mL).

### AcSDKP measurements

We analyzed urine AcSDKP concentrations using a competitive enzyme immunoassay kit (SPI-BIO, Massy, France), according to the manufacturer’s instructions. Urine AcSDKP was normalized with the urine creatinine level^15^.

### RNA extraction and miR array analysis

Frozen kidney tissues were first placed on the RNAlater^®^-ICE (Life technologies) for 16 h at −20 °C before the subsequent homogenization process. Total RNA was isolated using the miRNeasy kit (Qiagen) following the manufacturer’s instructions and was quantified with a Nanodrop spectrophotometer (ND-1000, Nano drop Technologies, DE, USA). The input for the Agilent miR labeling system was 100 ng total RNA. Dephosphorylated and denatured total RNA was labeled with cyanine 3-pCp and subsequently hybridized to the Agilent mouse miR microarray release version 15 using the miR Complete Labeling and Hyb Kit (Agilent Technologies, Inc., Santa Clara, CA, USA). Following hybridization for 20 h, the slides were washed with a Gene Expression Wash Buffer Kit (Agilent) and measured using an Agilent Scanner G2565BA. Agilent Feature Extraction Software (version 9.5.1) and GeneSpring GX software (version 12.5, Agilent) were used for data processing, analysis, and monitoring.

### RNA isolation and qPCR

Complementary DNA (cDNA) was generated by a miScript II RT kit (Qiagen) using the hiSpec buffer method. miR expression was quantified using a miScript SYBR Green PCR Kit (Qiagen), with 3 ng of complementary DNA. The primers for quantifying miR-29a-3p, miR-29b-3p, miR-29c-3p, miR-29a-5p, miR-29b-5p and miR-29c-5p were miScript primer assays pre-designed by Qiagen. The mature miR sequences were 5′UAGCACCAUCUGAAAUCGGUUA for miR-29a-3p, 5′ UAGCACCAUUUGAAAUCAGUGUU for miR-29b-3p, 5′UAGCACCAUUUGAAAUCGGUUA for miR-29c-3p, 5′ACUGAUUUCUUUUGGUGUUCAG for miR-29a-5p, 5′GCUGGUUUCAUAUGGUGGUUUA for miR-29b-5p and 5′UGACCGAUUUCUCCUGGUGUUC for miR-29c-5p. All experiments were performed in triplicate, and Hs_RNU6-2_1 (Qiagen) was utilized as an internal control. The primers to quantify miR-let7b-5p, miR-let7b-3p, miR-let7c-3p, miR-let-7c-5p, miR-let7f-3p, miR-let7g-3p, and miR-let-7i-3p were designed by Qiagen. The mature sequences were UGGAAGACUUGUGAUUUUGUUGU for miR-let7b-5p, CAACAAGUCACAGCCAGCCUCA for miR-let7b-3p, CUGUACAACCUUCUAGCUUUCC for miR-let7c-3p, 5′UGAGGUAGUAGGUUGUAUGGUU for miR-let-7c-5p, CUAUACAAUCUAUUGCCUUCCC for miR-let7f-3p, ACUGUACAGGCCACUGCCUUGC for miR-let7g-3p and CUGCGCAAGCUACUGCCUUGCU for miR-let-7i-3p, respectively.

### Transfection

The HMVECs were transfected with 100 nM of antagomir for miR-29a, miR-29c (Fasmac, Japan), miR-29b inhibitor (Qiagen), or mimetics for miR29s (29a-3p: UAGCACCAUCUGAAAUCGGUUA, 29b-3p: UAGCACCAUUUGAAAUCAGUGUU, 29c-3p: UAGCACCAUUUGAAAUCGGUUA) using Lipofectamine 2000 transfection reagent (Invitrogen, Carlsbad, CA, USA), according to the manufacturer’s instructions. The mimic and antagomir of miR-let-7b and c were purchased from Invitrogen, Carlsbad, CA, USA. The transfection studies (i.e., antagomir, mimic of miR-let-7b and miR-let-7c; antagomir, mimic of miR-29a, miR-29b and miR-29c, in the HMVECs at a 100 nM concentration) were also performed using Lipofectamine 2000 transfection reagent. For neutralizing IFN-γ experiment, HMVEC were transfected with antagomir of miR-29s (a + b + c), then the cells were treated with or without neutralizing IFN-γ (4 μg/ml) for 72 hours, cells were harvest for western blot or qPCR analysis.

### Luciferase assay

For the luciferase assay, to analyze the activity of the 3′UTR in human DPP-4, we cloned the fragment of human DPP-4 3′UTR sequence by PCR with the primer set (Fw: ATAGAGCTCAATAGCTAGCAGCACAGCACACCAAC Rev: ATATCTAGA GTGTCCATATGCCAGTGCGGTTTAGG) and BAC clone human RP11 178A14 as a template. The purified PCR fragment and pmirGLO Dual-Luciferase miR Target Expression Vector (Promega) were enzyme digested (Sac-1 and Xba-1), purified, and ligated (Thermo Fisher Scientific, Waltham, MA). The sequence of DPP-4 3′UTR was confirmed, and the amplified vector DNA (300 ng/well in a 12-well plate) was transfected into HMVEC cells. In the presence and absence of TGF-β2, AcSDKP was stimulated (100 nM in final concentration); the transcript in the transcriptional activity was evaluated with the Dual-Luciferase^®^ Reporter (DLR™) Assay System (Promega) in triplicate samples.

### Statistical analysis

The data are expressed as the means ± s.e.m. The one-way ANOVA Tukey test was performed to analyze the level of significance, which was defined as *P* < 0.05, if not specifically mentioned. GraphPad Prism software (Ver 3.0f) was used for the statistical analysis.

## Additional Information

**How to cite this article**: Srivastava, S. P. *et al*. Effect of Antifibrotic MicroRNAs Crosstalk on the Action of N-acetyl-seryl-aspartyl-lysyl-proline in Diabetes-related Kidney Fibrosis. *Sci. Rep*. **6**, 29884; doi: 10.1038/srep29884 (2016).

## Supplementary Material

Supplementary Information
