# Supplementary material for: Effect of Antifibrotic MicroRNAs Crosstalk on the Action of N-acetyl-seryl-aspartyl-lysyl-proline in Diabetes-related Kidney Fibrosis
Source: Sci Rep. 2016 Jul 18;6:29884. doi: 10.1038/srep29884 (PMC4947922; doi:10.1038/srep29884)
Supplement: Supplementary Information [file srep29884-s1.doc]

**Effect of Antifibrotic MicroRNAs Crosstalk on the Action of N-acetyl-seryl-aspartyl-lysyl-proline in Diabetes-related Kidney Fibrosis**

**Swayam Prakash Srivastava^1)^, Sen Shi^1)^, Megumi Kanasaki^1)^, Takako Nagai^1)^,** **Munehiro Kitada^1,2)^, Jianhua He^1)^, Yuka Nakamura^3)^, Yasuhito Ishigaki^3)^, Keizo Kanasaki^1,2)*^, Daisuke Koya^1,2)*^**

^1^Department of Diabetology & Endocrinology, Kanazawa Medical University, Uchinada, Ishikawa, Japan 920-0293

^2^Division of Anticipatory Molecular Food Science and Technology, Kanazawa Medical University, Uchinada, Ishikawa, Japan 920-0293

^3^Medical Research Institute, Kanazawa Medical University, Uchinada, Ishikawa, Japan 920-0293

***: Co-corresponding authors**

Address correspondence to:

Keizo Kanasaki

E-mail: [kkanasak@kanazawa-med.ac.jp](mailto:kkanasak@kanazawa-med.ac.jp)

or

Daisuke Koya

E-mail: [koya0516@kanazawa-med.ac.jp](mailto:kkanasak@kanazawa-med.ac.jp)

Department of Diabetology & Endocrinology

Kanazawa Medical University

Uchinada, Ishikawa 920-0293, Japan

TEL: 81-76-286-2211(Ex3305)

FAX: 81-76-286-6927

**Supplementary Information**

**Figure S1**

**Figures S1:** **Physiological characteristics and fibrogenic markers in non-diabetic and diabetic mice of CD-1 and 129Sv strains.** The induction of diabetes in CD-1 and 129Sv were according to well established protocol and has given in the method section. After 24 weeks, n=5 in each data set were analyzed for **A:** Blood glucose. **B:** Body weight. **C:** Kidney weight (mg/g body weight). **D:** Cystatin C (pg/ml) level. **E:** Polyoligopeptidase (POP) activity in non diabetic and diabetic mice kidney of CD-1 and 129Sv strains**. F:** Western blot analysis of thymosin β4, and the angiotensin converting enzyme N terminal (ACE-N) and ACE-C terminals in the non-diabetic and diabetic kidneys of the CD-1 and 129Sv mice strains. Representatives from 5 blots are shown here. The peptide AcSDKP is synthesize from thymosin β4 by the enzymatic activity of POP. It is degraded by ACE in the plasma. Therefore level of AcSDKP is regulated by POP and ACE activity and is tightly regulated. **G-H:** Densitometry analysis of the ACE-N and ACE-C terminal. The data were normalized by β-actin. **I:** ACE activity in the non-diabetic and diabetic kidneys of the CD-1 and 129Sv mice strains. **J-L:** Immunofluorescence analysis was performed by fluorescence microscopy in non-diabetic and diabetic mice kidney of CD-1 and 129Sv strain. **J:** CD31/α-SMA (CD31-red, α-SMA-green); **K:** CD31/TGFβR1 (CD31-Red, TGFβR1-Green); **L:** FSP-1/DPP-4 (FSP-1-red rhodamine, DPP-4-green FITC)**.** Merged pictures are shown. The original magnification was 400x. Scale bar: 50µM in each panel. The data are expressed as the means ± s.e.m and are included in the graph. The diabetic mice were designated as DM in the figure.

**Figure S2**

**Figure S2:** Immunofluorescence analysis was performed by fluorescence microscopy in the kidney of non-diabetic control and diabetic and AcSDKP treated diabetic CD-1 mice **A:** α-SMA/DPP-4 (α-SMA-Red, DPP-4-green); **B:** FSP-1/DPP-4 (FSP-1-red rhodamine, DPP-4-green FITC), **C:** TGFβR1/DPP-4 (TGFβR1-Red, DPP-4-Green); Merged pictures are shown. The original magnification was 400x. Scale bar: 50µM in each panel. **D.**  Measurement of Cystatin C level in control, diabetes and AcSDKP treated diabetic CD-1 mice

**Figure S3**

**Figure S3: AcSDKP inhibits DPP-4-associated TGFβ signaling in the HMVEC cells.** **A-D:** Western blot analysis and quantification of DPP-4 and TGFβ signaling in AcSDKP treated HMVEC with or without TGFβ2. Representative blots from 4 independent experiments are shown. **E:** Quantitative analysis of DPP-4 mRNA expression by real-time PCR using specific primers in the presence of TGFβ2 in HMVECs, n=5 in each data set were analyzed. **F:** Luciferase assay analysis in transfected HMVECs containing the DPP-4 mRNA 3’UTR construct; fold change in the luciferase/renilla activity, TGFβ2-treated, and AcSDKP-treated cells, n=6 in each data set were analyzed. Data are expressed as the mean±s.e.m. and are shown in the graph.

**Figure S4**

**Figure S4: AcSDKP restores the downregulated status of miR-29s and linagliptin restores downregulated status of miR-let-7s in the TGFβ2 stimulated HMVECs. A-C:** qPCR analysis of miR-29s in the control, TGFβ2, AcSDKP stimulation in the TGFβ2 in the HMVECs, n=6 in each data set were analyzed. **A:** miR-29a-3p. **B:** miR-29b-3p. **C:** miR-29c-3p. **D-I:** qPCR analysis of miR-let-7s in the control and linagliptin with or without TGFβ2 in the HMVECs, n=6 in each data set were analyzed. **D:** Fold change in miR-let-7b-3p. **E:** miR-let-7b-5p. **F:** miR-let-7c-3p. **G:** miR-let-7f-3p. **H:** miR-let-7g-3p and **I:** miR-let-7i-3p. Hs_RNU6 was used as internal control. Data are expressed as the mean±s.e.m. and are shown in the graph.
